# Association of phenylthiocarbamide perception with anthropometric variables and intake and liking for bitter vegetables

**DOI:** 10.1186/s12263-022-00715-w

**Published:** 2022-07-27

**Authors:** Marta Trius-Soler, Paz A. Bersano-Reyes, Clara Góngora, Rosa M. Lamuela-Raventós, Gema Nieto, Juan J. Moreno

**Affiliations:** 1 Department of Nutrition, Food Sciences and Gastronomy, XIA, Faculty of Pharmacy and Food Sciences, University of Barcelona, 08028 Barcelona, Spain; 2 INSA-UB, Instituto de Investigación en Nutrición y Seguridad Alimentaria, Universidad de Barcelona, 08921 Santa Coloma de Gramenet, Spain; 3 CIBER Fisiopatología de la Obesidad y Nutrición (CIBEROBN), Instituto de Salud Carlos III, 28029 Madrid, Spain; 4 Department of Food Technology, Food Science and Nutrition, Faculty of Veterinary Sciences, Regional Campus of International Excellence “Campus Mare Nostrum”, Espinardo, 30071 Murcia, Spain

**Keywords:** Recognition threshold, Non-tasters, Super-tasters, Body mass index, Brassicaceae, Bitter taste

## Abstract

Phenylthiocarbamide (PTC) sensitivity, a sensory trait mediated by the bitter taste receptor 38 (TAS2R38), has been described as a promising biomarker of health status or disease risk. The aim of this cross-sectional study was to evaluate the influence of PTC phenotypes on (1) individual anthropometric and clinical history variables; (2) other basic taste recognition thresholds (RTs), and (3) the hedonic perception and habitual intake of *Brassicaceae* vegetables in a young adult population (18.9 ± 1.7 years old). The PTC phenotype was determined by the quantitative measure of the PTC recognition threshold (non-tasters, 24.1%; tasters, 52.3%; and super tasters, 23.6%). No significant differences in smoking habits, oral and nasal disorders, family antecedents of diseases related to metabolic syndrome, and *Brassicaceae* vegetable hedonic perception and consumption were found between the PTC phenotype groups. The average BMI of super-taster females and males was significantly lower compared to non-tasters. In addition, the PTC taster status was a predictor of lower scores for other basic taste RTs. Overall, the defined PTC super-taster cohort could be differentiated from the non-tasters by variables related to weight control such as BMI and sucrose RT.

**Supplementary Information:**

The online version contains supplementary material available at 10.1186/s12263-022-00715-w.

## Introduction

Mammals have complex sensory transduction pathways to distinguish the quality and safety of food, and they can differentiate between at least five basic tastes: sweet, bitter, salty, sour and umami. The chemosensory perception of taste is complemented by the olfactory system [1]. In particular, bitter taste is believed to be a protective sensory response to prevent the ingestion of potentially toxic compounds, although not all bitter foods are toxic [2]. The genetic background of sensitivity to bitter taste has been extensively researched. To date, the majority of genotype-phenotype studies in this field have focused on the polymorphisms of the gene encoding the bitter taste receptor 38 (T2R38), which are responsible for the different phenotypes of people who are insensitive or taste-blind (non-tasters), moderately sensitive (tasters) and highly sensitive (super-tasters) to certain bitter substances [3, 4]. This discovery was made in 1931 with an artificial compound, phenylthiocarbamide (PTC) [5]. It is now known that phenotypic variation in perception of PTC bitterness, including super-tasting, can be explained to a large extent, but not fully, by T2R38 polymorphisms [6, 7].

The phenotypic variation in sensitivity to PTC and structurally related compounds has been associated with indicators of obesity [8–10], as well as with an unequal response to a weight loss intervention [11]. In particular, non-tasters females have been reported to have a higher body mass index (BMI) than tasters and super-tasters [10, 12]. However, only a few studies have analyzed BMI as a continuous variable or its relationship with PTC/PROP sensitivity [13–15].

Interestingly, super-tasters are reported to have a higher density of fungiform papillae on the tongue compared to tasters and non-tasters [4]. Furthermore, high sensitivity to bitter taste has been linked to an enhanced perception of sweetness [16]. However, the relationship between bitter and the other basic taste sensitivities still requires more in-depth research.

The synthetic compound PTC contains a thiocyanate (N-C=S) group that is also found in 6-n-propylthiourail (PROP) and isothiocyanates. The main dietary sources of isothiocyanates belong to the *Brassicaceae* family (e.g., cruciferous vegetables, mustard, and arugula) [17, 18] and their intake has been hypothesized to be lower among PTC tasters in some studies [19–21]. Although a wide range of factors can influence food choices and preferences, taste is held to one of the most important [22, 23]. Age and sex are reported to modify the relationship between food choices and PROP/PTC taster status [4].

Understanding the underlying characteristics of the PTC phenotype is a critical first step in harnessing its usefulness as a biomarker of health and disease. Therefore, the present study aimed to evaluate if PTC taste status has an influence on (1) individual anthropometry and clinical history; (2) the basic taste recognition thresholds (RTs) and estimated total taste acuity; (3) and the consequent possible differences in liking and/or consumption of vegetables with a PTC-related bitter taste. This cross-sectional study was carried out in a large Spanish young adult cohort of college students and represents the first investigation of these research questions in this specific population.

## Results

### Anthropometric and clinical differences between PTC super-tasters, tasters, and non-tasters by sex

The differences in anthropometric and clinical variables between PTC non-tasters, tasters, and super-tasters by sex are shown in Table 1. Among all the participants, 24.1% were non-tasters, 52.3% were tasters, and 23.6% were super-tasters. By sex, 22.2% of women and 27.5% of men were non-tasters, 55.7% and 46.5% were tasters, and 22.2% and 26.1% were super-tasters, respectively.

**Fig. 1:**
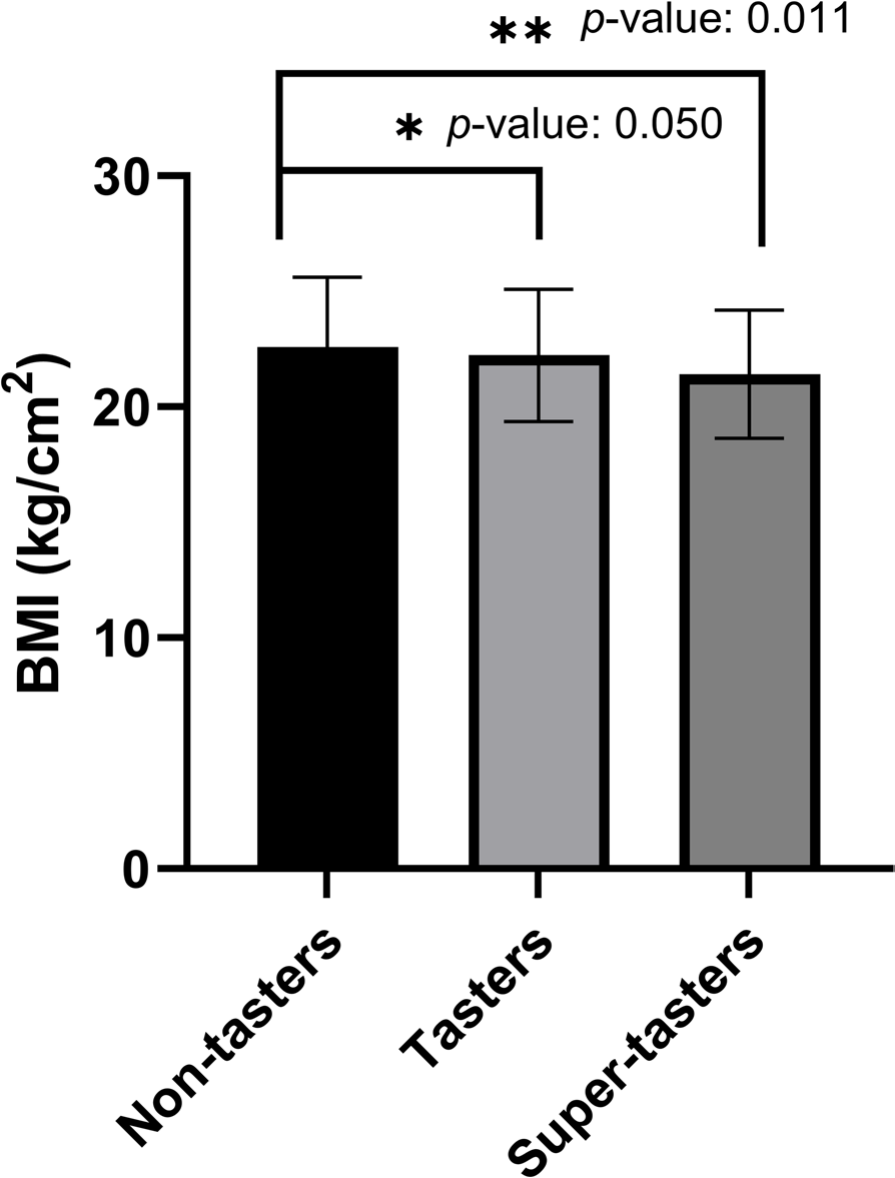
Body mass index **(**BMI) (mean ± SD) differences between phenylthiocarbamide taster status groups (*n* = 381)

The three well-established PTC phenotypes did not differ significantly in tobacco smoking habits, oral and nasal disorders, and first- and second-degree metabolic syndrome-related diseases. Among women, super-tasters had a significantly lower mean BMI compared to non-tasters, and among men, super-tasters also had the lowest mean BMI (Fig. 1, Table 1).

**Table 1:** Anthropometric and clinical differences between phenylthiocarbamide taster status groups by sex

	Sex	Non-tasters (***n*** = 94)	Tasters (***n*** = 204)	Super-tasters (***n*** = 92)	***p-***value
Smokers, *n (%)*	F	8 (14.6)	22 (15.9)	8 (14.6)	0.955
	M	12 (30.77)	17 (25.8)	7 (18.9)	0.492
Caries, *n (%)*	F	36 (65.5)	82 (59.4)	29 (52.7)	0.397
	M	19 (48.7)	40 (60.6)	19 (51.4)	0.436
Missing teeth, *n (%)*	F	2 (3.6)	4 (2.9)	1 (1.8)	0.845
	M	1 (2.6)	6 (9.1)	3 (8.1)	0.431
Sinusitis, *n (%)*	F	3 (5.5)	10 (7.3)	2 (3.6)	0.623
	M	4 (10.3)	3 (4.6)	4 (10.8)	0.411
Rhinitis, *n (%)*	F	5 (9.1)	9 (6.5)	2 (3.6)	0.507
	M	5 (12.8)	2 (3.0)	3 (8.1)	0.159
BMI, *kg/m*^*2*^	F	21.8 ± 2.9^a^	21.5 ± 2.6^ab^	20.7 ± 2.7^b^	**0.043**
	M	23.6 ± 2.8^ab^	23.6 ± 2.9^a^	22.4 ± 2.6^b^	0.099
BMI diagnosis, *n (%)*					
*Underweight*	F	4 (7.6)	14 (10.4)	10 (18.5)	0.133
*Normal weight*		39 (73.6)	104 (77.0)	41 (75.9)	
*Overweight-obese*		10 (18.9)	17 (12.6)	3 (5.6)	
*Underweight*	M	1 (2.6)	1 (1.5)	3 (8.3)	0.351
*Normal weight*		28 (73.7)	45 (68.2)	26 (72.2)	
*Overweight-obese*		9 (23.7)	20 (30.3)	7 (19.4)	
Family antecedents, n (%)					
*Diabetes*	F	16 (29.1)	55 (39.9)	20 (36.4)	0.374
*Hypertension*		14 (25.5)	58 (42.0)	24 (43.6)	0.071
*Obesity*		13 (21.8)	29 (21.0)	9 (16.4)	0.723
*Diabetes*	M	12 (30.8)	15 (23.1)	11 (29.7)	0.628
*Hypertension*		13 (33.3)	26 (39.4)	18 (46.7)	0.390
*Obesity*		9 (23.1)	14 (21.2)	3 (8.1)	0.170

*BMI* body mass index, *F* females, *M* males

Continuous variables are expressed as mean ± SD and categorical variables as n (%). Statistical analyses were carried out using the Kruskal-Wallis rank-sum test (post-hoc Dunn test) when comparing quantitative variables. A chi-square test was used for categorical variables. Different letters indicate significant differences (*p-*value <0.05)  among groups. Values shown in bold are statistically significant *p-*value < 0.05

One participant had missing data on family antecedents of diabetes, and three, missing data on BMI

### Influence of PTC tasting status on taste recognition thresholds

The influence of PTC tasting status on basic taste RTs, as well as on the total taste score (TTS), was investigated. Among women, the PTC super-taster group had significantly lower RTs for sucrose and sinigrin as well as TTS compared to the non-tasters. Among males, basic taste RTs were systematically lower in super-tasters compared to tasters and non-tasters. Overall, a higher proportion of PTC super-tasters could detect saccharin bitterness (91.9% of super-taster males and 74.6% of super-taster females). Quinine was the only tested substance without significant differences in RT between the PTC super-tasters and non-tasters. Thus, individuals who were most sensitive to PTC bitterness were also the most sensitive to the other basic tastes. These results were more pronounced in the male cohort *(*Table 2*).*

**Table 2:** Influence of phenylthiocarbamide taster status on basic taste recognition thresholds, total bitter score, and total taste score of the studied taste stimuli by sex

	Sex	Non-tasters	Tasters	Super-tasters	***p***-value
Sucrose	F	5.7 ± 1.1^a^	5.2 ± 1.2^a^	4.7 ± 1.2^b^	**< 0.001**
	M	5.1 ± 1.7^a^	5.2 ± 1.3^a^	4.1 ± 1.2^b^	**< 0.001**
Monosodium glutamate	F	2.8 ± 1.0^a^	2.7 ± 0.9^a^	2.5 ± 0.8^a^	0.347
	M	3.1 ± 1.4^a^	2.7 ± 0.9^ab^	2.3 ± 0.5^b^	0.062
Sodium chloride	F	4.6 ± 1.1^a^	4.6 ± 0.9^a^	4.3 ± 0.8^a^	0.106
	M	4.6 ± 1.1^a^	4.6 ± 0.9^a^	4.1 ± 0.6^b^	**0.007**
Citric acid	F	3.5 ± 1.1^a^	3.4 ± 1.0^a^	3.2 ± 1.2^a^	0.204
	M	3.5 ± 1.4^a^	3.4 ± 1.2^a^	2.5 ± 1.3^b^	**0.001**
Quinine	F	3.1 ± 1.3^ab^	3.0 ± 1.2^a^	2.9 ± 0.9^b^	0.514
	M	3.2 ± 1.7^ab^	3.3 ± 1.2^a^	2.6 ± 0.9^b^	**0.031**
Sinigrin	F	3.7 ± 0.7^a^	3.0 ± 0.9^b^	2.9 ± 1.0^b^	**< 0.001**
	M	3.2 ± 1.1^a^	3.3 ± 0.8^a^	2.3 ± 1.2^b^	**< 0.001**
TTS	F	0.504 ± 0.108^a^	0.443 ± 0.098^b^	0.399 ± 0.097^c^	**< 0.001**
	M	0.487 ± 0.162^a^	0.462 ± 0.104^a^	0.326 ± 0.106^b^	**< 0.001**
Saccharin	F	30 (52.6)	95 (69.3)	41 (74.6)	**0.030**
*(bitterness detected)*	M	17 (39.5)	46 (69.7)	34 (91.9)	**< 0.001**

*F* females, *M* males, *TTS* total taste score

Quantitative variables are expressed as mean ± SD and categorical variables as n (%). Statistical analyses were undertaken using the Kruskal Wallis rank-sum test (post-hoc Dunn test) when comparing quantitative variables. A chi-square test was used for categorical variables. Different letters indicate significant differences (*p*-value <0.05) among groups. Values shown in bold are statistically significant, *p*-value < 0.05

### Influence of PTC tasting status on the liking and consumption of bitter vegetables

Participants were asked if they like and consume cruciferous vegetables, mustard, or bitter leaves (endive and arugula). After the statistical analysis, no differences were found between the PTC tasting groups stratified by sex (Table 3). In the three groups, 36.4–61.8% of the individuals affirmed that they liked and consumed bitter vegetables. Moreover, the majority liked and consumed one or two out of the three options, resulting in a mean sum of bitter food intake of 1.5 ± 0.8 out of a possible total score of 3.

**Table 3:** Differences in the proportions of participants liking and consuming bitter foods among phenylthiocarbamide taster status groups

	Sex	Non-tasters	Tasters	Super-tasters	***p***-value
Cruciferous	F	33 (58.9)	82 (60.7)	34 (61.8)	0.951
	M	20 (45.5)	27 (42.2)	17 (50.0)	0.759
Mustard	F	24 (42.7)	72 (53.3)	29 (53.7)	0.379
	M	16 (36.4)	26 (41.3)	14 (40.0)	0.875
Bitter leaves	F	29 (50.9)	77 (56.2)	24 (43.6)	0.281
*(Endive and arugula)*	M	19 (44.2)	29 (44.6)	19 (54.3)	0.597
Sum of bitter food intake	F	1.4 ± 0.8	1.5 ± 0.9	1.6 ± 0.8	0.444
	M	1.3 ± 0.8	1.3 ± 0.8	1.4 ± 0.9	0.739

*F* females, *M* males

Categorical variables as n (%). A chi-square test was applied

### Multivariate analysis of the inter-individual differences across stimuli

**Fig. 2:**
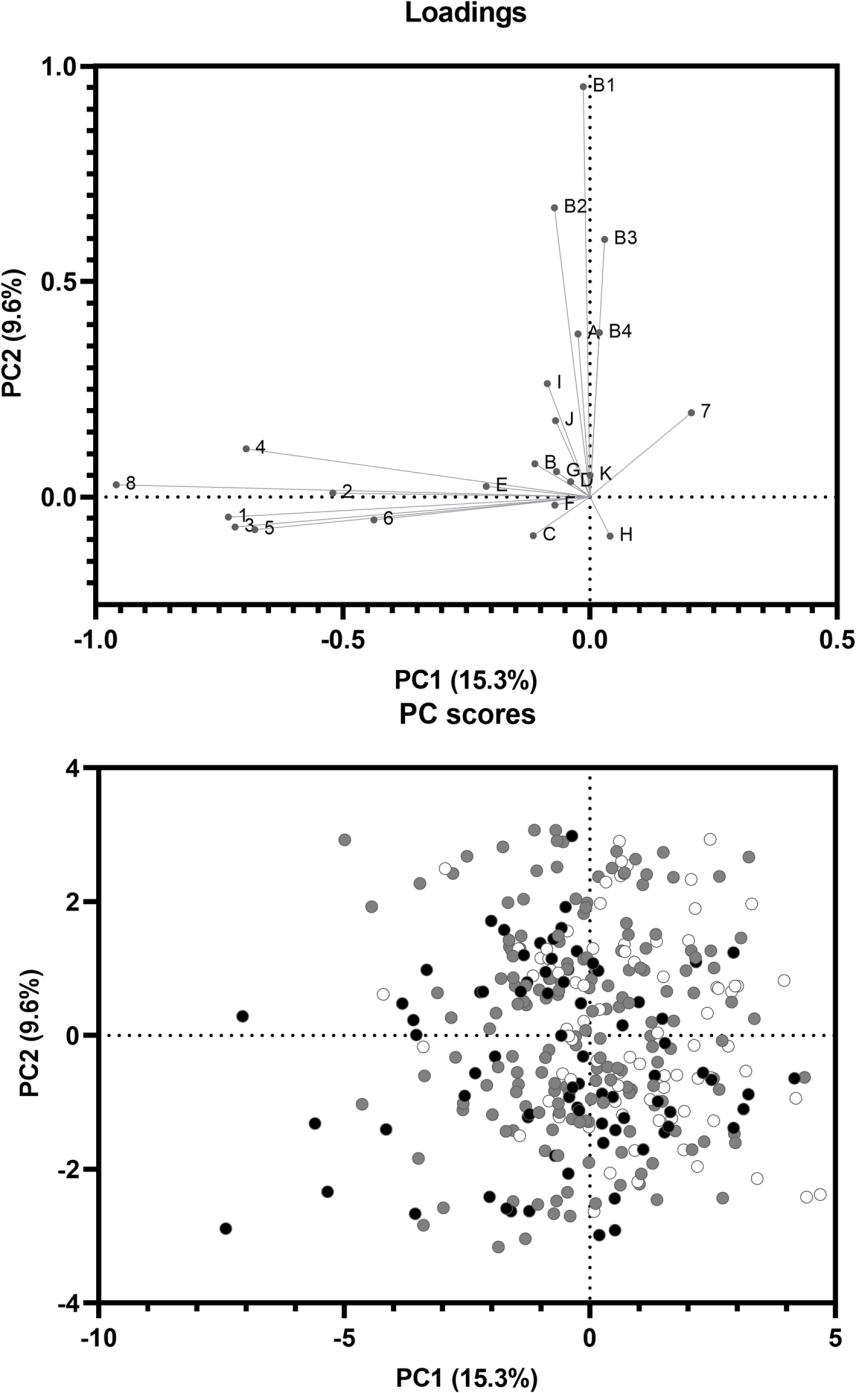
Principal Component Analysis (PCA) of individual characteristics, taste sensitivity, and liking and consumption of selected bitter vegetables. Black points indicate the non-taster phenylthiocarbamide (PTC) group; gray points indicate the taster PTC group, and white points indicate the super-taster PTC group. A, sex; B, age; C, smoking habit; D, body mass index; E, sinusitis; F, rhinitis; G, caries; H, missing teeth; I, family antecedents of diabetes; J, family antecedents of hypertension; K, family antecedents of obesity; B1, liking and total consumption of bitter food; B2, liking and consumption of cruciferous vegetables; B3, liking and consumption of endive and arugula; B4, liking and consumption of mustard; 1, sucrose recognition threshold; 2, monosodium glutamate recognition threshold; 3, sodium chloride recognition threshold; 4, citric acid recognition threshold; 5, quinine recognition threshold; 6, sinigrin recognition threshold; 7, saccharin bitterness perception; 8, total taste score

To understand how the different RTs, clinical history, and liking and consumption of bitter vegetables of individuals are correlated, a PCA (Principal Component Analysis) plot of the analytical data was generated, which reduced the dimension of the dataset while retaining as much information from the data as possible. A PCA was applied to the matrix of 335 subjects × 23 parameters. Principal components (PCs) were computed from participant inter-individual differences in each PCA model. In the PCA of the Imax matrix, the first two PCs were selected. Fig. 2 shows the bi-dimensional plot of PC 2 vs. PC 1. Vectors of the original variables are plotted according to their factor loadings. The PCA biplot accounted for about 24.9% of the variation within the data set. The first major observations are that all stimuli are distributed on the negative side of PC 1 (15.3% of variation) and was strongly correlated with TTS (values close to − 1). A negative loading in PC 1 grouped the RTs close together, indicating that the RT scores are positively correlated. Another interesting observation is that bitter food liking and consumption are located on the positive side of PC 2, explaining 9.6% of the total inter-variability.

On the other hand, the PC score graph shows a tendency of data points separation between super-tasters and non-tasters of PTC, however it appears to be overlapped. From left to right and from bottom to top, it can be observed that PTC super-tasters are located more towards the bottom-left quadrant of the graph, while non-tasters are near the top-right quadrant. Tasters are located between the two other PTC taster status groups.

## Discussion

T2R38 is a member of the T2R bitter taste receptor gene family, which in humans includes 25 functional genes and 11 pseudogenes with several genetic signatures of natural selection [24–26]. The association between T2R38 gene variation and sensitivity to thiourea bitterness has been well characterized by numerous research teams [27, 28]. Therefore, the present study evaluated the applicability of this phenotypic variation in PTC perception as a biomarker of health-related factors, global taste sensitivity and hedonic perception and intake of *Brassicaceae* vegetables. Interestingly, it was observed that PTC tasting status is related to other basic taste RTs and BMI. Conversely, PTC RTs were not correlated with differences in hedonic perception and consumption of bitter vegetables.

A primary aim of the current work was to identify if health-related factors had a variable distribution among PTC taster status groups, as might be expected according to current evidence. For example, polymorphisms of the TAS2R38 gene are linked to significant differences in the ability of upper respiratory cells to clear and kill bacteria [29], and consequently may be involved in susceptibility to upper respiratory infection and recalcitrant sinusitis [30]. However, our results did not show a different incidence of sinusitis and rhinitis between PTC non-tasters, tasters, and super-tasters. Nor did PTC non-tasters and tasters differ in smoking habits, in contrast with other studies that have observed effects of smoking on PTC thresholds [15, 31]. Regarding sex, although it has been suggested that a higher frequency of super-tasters are female [32], no differences were found in our cohort. These results might be due to the age range of the studied population, which supports the possibility that the pattern of sex difference can differ according to the age group.

In this cohort of healthy Spanish young adult college students, the super-tasters had a lower BMI than tasters and non-tasters. The same finding was also reported by Padiglia *et al*. (2010) in an Italian cohort of normal weight 20–29-year-old females and males [12]. Studies carried out in older subjects also report that non-tasters are more likely to have a higher BMI than super-tasters [10, 15, 33]. However, other studies have not found any evidence of a relationship between BMI and PTC taster status [11, 34].

Regarding T2R38 phenotypes, Tepper *et al*. (2008) found that a high PROP sensitivity was associated with a lower BMI when demographic variables and the PROP dietary restraint interaction term were included in the model in females but not males, describing PROP as a marker for susceptibility to weight gain only in females [14]. On the other hand, the PROP detection threshold was positively correlated with BMI in both obese and normal weight groups in another study [13]. Additionally, the PROP phenotype was described as a better predictor of variation in body weight compared to the T2R38 genotype [14].

Interestingly, the results of the present study showed that basic taste RTs of the PTC non-tasters were higher compared to the super-tasters for sucrose and sinigrin, and they also had a higher TTS. In PTC super-taster males, hypergeusia was also observed for other evaluated stimuli, and lower RTs were recorded for sodium chloride (NaCl), citric acid and quinine. These findings are consistent with the results of previous studies based on taste thresholds [35, 36] and suprathreshold intensity scaling [2, 6]. Anatomical data also support these differences between PTC taster groups, as super-tasters reportedly have more fungiform papillae and taste buds than non-tasters [37]. Although the data about PTC tasting refer specifically to the perceived intensity of bitterness, differences in other RTs between the PTC taster groups are also of interest, as they reflect the ability to distinguish between the five basic tastes.

PTC non-taster status has been associated with a higher accumulation of adiposity [10, 14], and unhealthy food preferences and dietary habits (e.g., a higher consumption and acceptance of fat) [33, 38] that favor the development of chronic non-communicable diseases (e.g., diabetes, obesity) and certain types of cancer. Interestingly, our findings suggest that these health-impacting tendencies can also be related to a lower sensitivity to other basic tastes involved in calory intake, such as sweetness, as reported previously by our group [39], and umami. Furthermore, the effects of weight loss interventions or dietary restraint on BMI may vary according to the PTC phenotype [11, 40]. Intestinal type 1 taste receptors (T1Rs), which are also responsible for sweet and umami tastes, are associated with the secretion of gut hormones, interfering with sodium-dependent glucose transport after sugar ingestion [41]. Research on the physiological implications of the gustatory function is currently growing, and new data are emerging on its ability to predict health status and the role of taste sensitivity in disease prognosis [39, 42, 43]. These discoveries contribute to the body of evidence supporting the hypothesized relationship between PTC taste perception and body weight homeostasis in young healthy individuals.

The discovery of extra-oral T2Rs in several metabolically active tissues has generated intense interest in their physiological significance and potential health impact [44]. T2Rs, which are expressed in enteroendocrine cells, can be involved in nutrient-gut interactions that modulate the secretion of gut hormones such as ghrelin, cholecystokinin, and glucagon-like peptide 1, thereby influencing gastrointestinal motility, appetite, and glycemia [41]. Consequently, a T2R38 genotype could have important consequences for weight homeostasis involving some of these mechanisms, as suggested recently [45, 46]. These bitter taste receptors in the intestinal tract were found to be upregulated in overweight/obese subjects, indicating that sensory receptors are involved in diet-related weight increases [8, 47]. Interestingly, T2R38, at both RNA and protein levels, has been recently described in human adipocytes, with higher expression levels in the adipose tissue of obese compared to lean individuals. Moreover, the in vitro stimulation of T2R38 by PROP induced a decrease in lipid accumulation, suggesting that T2R38 expression can modulate adipocyte functions. T2R38 gene variants did not influence the expression levels of the receptor [48].

Although neither PTC nor PROP are found in foods, other thiourea-containing compounds such as glucosinolates are present in cruciferous vegetables. In the present study, PTC taster status did not affect the liking or consumption of cruciferous vegetables, mustard, and bitter leaves. Scientific evidence for the relationship between the ability to taste PTC and reported preferences for bitter vegetables is inconsistent. Food choices are influenced by multiple factors, making it difficult to predict a habitual diet if physical activity, cultural practices, socioeconomic status, and food access are not taken into consideration. Current results indicate that a single marker, be it phenotypic or genotypic, is insufficient to fully characterize orosensory responses related to diet and health [32, 49].

This study was carried out in a mixed-sex cohort aged 18–30 years representative of the Spanish college student population. The methodology applied was low cost and non-invasive, and the sample size was large in comparison with previous studies. Height and weight were measured to eliminate the risk of false self-reporting. Furthermore, a quantitative determination of basic taste sensitivity was performed, in contrast with the majority of studies in this field, which are based on qualitative/semi-quantitative estimations. However, the study also has some limitations. First, BMI was used as a marker of obesity, although this has been questioned as a criterion for classifying fatness in college athletes and non-athletes [50]. Second, the estimation of RT values by same-different task methodology (discriminating test type) could have been more precise. Indeed, the lack of standardized screening methods for PTC/PROP taste sensitivity hinder valid across-study comparisons, although 3-alternative forced choice (3-AFC) with reversal might be the more adequate method, it would have been at the same time too tedious and tiring to the participants due to the high number of solutions tasted. Finally, few data about dietary intake were collected (e.g., no food frequency questionnaires or 24-h records) and data on bitter vegetable liking and consumption were only descriptive, based on dichotomous questions (yes/no).

## Conclusion

The relationship of the three well-known phenotypes of PTC perception with anthropometric and clinical history variables, taste acuity determined by measuring basic taste RTs, and differences in the liking and consumption of *Brassicaceae* bitter vegetables was investigated in a large sample of young adults as part of a cross-sectional study. In this healthy homogeneous young population, only PTC super-tasters were differentiated from non-tasters and tasters by BMI, a factor related to weight control. Furthermore, PTC non-taster status was able to predict higher scores (low sensitivity) in other basic taste RTs and TTS. On the contrary, PTC taster status was not associated with liking perception and/or consumption of vegetables with a PTC-related bitter taste. Further investigations on super-tasting should be conducted to evaluate and confirm these associations and analyze the mechanisms involved.

## Material and methods

### Chemicals

Sucrose, monosodium glutamate (MSG), NaCl, citric acid, PTC, quinine, sinigrin and saccharine were supplied by Sigma Aldrich (St. Louis, MO, USA). Distilled water was used as the solvent to prepare the corresponding solutions.

### Study design and participants

A total of 403 students were recruited between October 2017 and April 2019. Participants older than 30 years were excluded from the analysis (*n* = 6). Missing PTC RT data was also an exclusion criterion (*n* = 7). Among the participants in the study, 357 were aged between 17 and 29 years (mean age: 18.9 ± 1.7 years), but other students with missing age data were included on the assumption they belonged to the same age range. Thus, the final cohort consisted of 390 young adults, all of whom were graduate students in Culinary and Gastronomic Sciences, Food Science and Technology, or Human Nutrition and Dietetics at the Torribera Campus of the University of Barcelona.

The research (Torribera Students Taste Study) was carried out according to the Declaration of Helsinki for Medical Research on Human Beings (WMA, 2001), and approved by the Ethics Committee of the University of Barcelona (Institutional Review Board: IRB 00003099). All the students were given information about the study, knew the objectives and benefits, accepted, and formalized their participation by signing the informed consent.

### Recognition threshold and stimulus concentrations

RT is the lowest concentration of a tastant that elicits a particular taste response [51]. The RT assessment methodology used in this study was a same-different task approach [52]. Participants were provided with successive sets of two samples. Each set contained one blank sample (water) and one stimulus sample (chemical dilutions). For each set, participants indicated if the samples tasted different and if they could recognize the taste. The regional stimulation was done by dropping 0.5 mL of sample at room temperature on the tip of the tongue for 5–10 s. Before tasting the next test solution, participants rinsed out their mouths with water and waited for 20 s.

Sets were presented in ascending concentrations (Table 4). The assay stopped when the participant correctly recognized the stimulus sample at a given concentration twice consecutively. The concentration at which the procedure stopped was considered the stimulus RT. We used a wide concentration range for each molecule, considering the bibliography about the topic [35, 53–55]. The order of sensory testing across taste qualities was the same among all participants and they were tested during the same test session. Before each new taste test, participants rinsed their mouths with water. Participants were asked not to chew gum, eat any product, or smoke for 2 h before the test.

**Table 4:** Concentrations of taste test solutions

	Sweet	Umami	Salty	Sour	Bitter		
Score	Sucrose (mM)	MSG (mM)	Sodium chloride (mM)	Citric acid (mM)	PTC (μM)	Quinine (μM)	Sinigrin (μM)
1	1.2	3.0	3.9	1.2	0.7	9.4	50
2	2.3	7.5	7.8	2.3	3.5	18.7	100
3	4.7	15.0	15.6	4.7	14	37.5	300
4	9.4	30.0	31.3	9.4	56.2	75	600
5	18.8	60.0	62.5	18.7	112.5	150	–
6	37.5	120.0	125.0	37.5	225	300	–
7	75.0	–	250.0	75.0	900	–	–
8	150.0	–	500.0	–	–	–	–

*MSG* monosodium glutamate, *PTC* phenylthiocarbamide

### Determination of PTC taster status

PTC taster status was determined on the theoretical assumption that the PTC phenotype is a marker of a variety of chemosensory experiences, including the RT [56]. Participants with a PTC RT score of 1 (≤ 0.7 μM) were classified as super-tasters; those with a score of 2 or 3 (3.5–14 μM) were classified as tasters; finally, those with a score higher than 3 (> 14 μM) were in the non-taster group. The three PTC taster status groups correspond to the three phenotypic groups with a known distribution in the Caucasian population (approximately 25% super-tasters, 50% tasters and 25% non-staters) [2]. In parallel, taster status distribution was studied by calculating the ratio of the PTC RT scores divided by the NaCl RT scores. The proportions of the generated groups were very similar, and the PTC taster groups obtained by the two approaches had a Spearman coefficient correlation of 0.896. Moreover, the cumulative RT frequency curve for PTC also showed a trimodal distribution (Supplementary Fig. 1), corresponding to the cut-off used. Although we cannot eliminate all classification errors, the three rational strategies came with a similar conclusion.

Elderly tasters are reported to have a higher mean PTC RT than young tasters [57]. However, few studies have used PTC RT as a method of classifying individuals into groups of PTC taster status [4], and limited data were available in the literature to establish PTC cut-off scores for the present study, considering the age of the participants and the method used for evaluating taste function.

### Predictor assessment: data collection

All participants filled in a brief structured self-reported questionnaire (paper) in person, which provided us with data about their clinical history and preference for and consumption of bitter vegetables. Accordingly, data about age, sex, smoking habits, discomfort in the mouth (caries and missing teeth) and nose-related complaints (sinusitis, rhinitis) were recorded. As only fifteen subjects had a smoking habit of more than 10 cigarettes/day, smoking habit was not a dividing factor in the analysis. First- and second-degree family histories of diabetes, hypertension, and obesity were also recorded. Liking and consumption of cruciferous vegetables, endives, arugula, and mustard were verified by asking a dichotomous question (yes/no). To establish perception of saccharin bitterness, the participants were administered a test solution (10 mM) and asked if they detected it or not.

Height and body weight of participants wearing light clothes and no shoes were measured following the International Standards for Anthropometric Assessments [58]. BMI was calculated as weight/height squared (kg/m^2^) and classified as “low weight” if < 18.5 kg/m^2^, “normal weight” if 18.5–24.9 kg/m^2^ and “overweight-obesity” if ≥25 kg/m^2^ [59].

### Statistical analysis

Statistical analyses were carried out to compare the PTC taster status groups of non-tasters, tasters, and super-tasters. The Kruskal-Wallis rank-sum test was applied for continuous variables, Dunn’s post-hoc correction for multiple comparisons, and the chi-square test was used to compare proportions of categorical variables among the PTC taster groups. Standardized PCA was applied, and PCs were selected based on a parallel analysis. Sex stratification analyses were performed to ascertain the impact of sex on the results.

Basic taste RTs were scaled in multiples of 1 standard deviation *(*Table 4*).* The TTS was calculated by adding the normalized RT scores for the five basic tastes and dividing by five, resulting in a new variable ranging from 0 to 1. To estimate the different RTs, the following tastants were used: sucrose for sweet; MSG for umami; NaCl for salty, and citric acid for sour. The score for the bitter RT was calculated by adding together the respective normalized scores obtained for the three bitter tastants (PTC, quinine, and sinigrin) and dividing by three, which resulted in a representative bitterness sensitivity score ranging from 0 to 1. Variables were normalized using Min-Max scaling to range the data into the same scale, following the general formula:

$${x}^{\prime }=\frac{x-\min (x)}{\max (x)-\min (x)}$$

Due to the large dataset and the small number of participants with missing information, no data imputation was applied in the statistical comparisons. However, the mean age was imputed for the PCA, resulting in a total analysed PCA sample of 335 participants. Statistical tests were two-sided, and *p*-values below 0.05 were considered significant. All statistical analyses were conducted using the Stata statistical software package version 16.0 (StataCorp, College Station, TX, USA). Data were visualized using GraphPad Prism 9 (GraphPad Prism Software, Inc. La Jolla, CA).

## Supplementary Information

**Additional file 1: **Cumulative frequency curves of the PTC recognition threshold scores.

## Data Availability

The datasets generated during and/or analyzed during the current study are available upon request to J.J. M.

## Abbreviations

BMI: Body mass index
WHO: World Health Organization
SD: Standard deviation
PTC: Phenylthiocarbamide
TTS: Total taste score
PROP: 6-n-propylthiourail
