# Supplementary material for: Association of phenylthiocarbamide perception with anthropometric variables and intake and liking for bitter vegetables
Source: Genes Nutr. 2022 Jul 27;17:12. doi: 10.1186/s12263-022-00715-w (PMC9331802; doi:10.1186/s12263-022-00715-w)
Supplement: Supplementary file 1 — Additional file 1: Cumulative frequency curves of the PTC recognition threshold scores. [file 12263_2022_715_MOESM1_ESM.docx]

**Association of phenylthiocarbamide perception with anthropometric variables and intake and liking for bitter vegetables**

Marta Trius-Soler^1-3^, Paz A. Bersano-Reyes^4^, Clara Góngora^1^, Rosa M. Lamuela-Raventós^1-3^, Gema Nieto^4^ and Juan J. Moreno^1-3*^

^1^ Department of Nutrition, Food Sciences and Gastronomy, XIA, Faculty of Pharmacy and Food Sciences, University of Barcelona, 08028 Barcelona, Spain.

^2^ INSA-UB, Instituto de Investigación en Nutrición y Seguridad Alimentaria, Universidad de Barcelona, 08921 Santa Coloma de Gramenet, Spain.

^3^ CIBER Fisiopatología de la Obesidad y Nutrición (CIBEROBN), Instituto de Salud Carlos III, 28029 Madrid, Spain.

^4^ Department of Food Technology, Food Science and Nutrition, Faculty of Veterinary Sciences, Regional Campus of International Excellence "Campus Mare Nostrum", Espinardo, 30071 Murcia, Spain.

*Correspondence author: e-mail address: jjmoreno@ub.edu; Tel: 934035818.


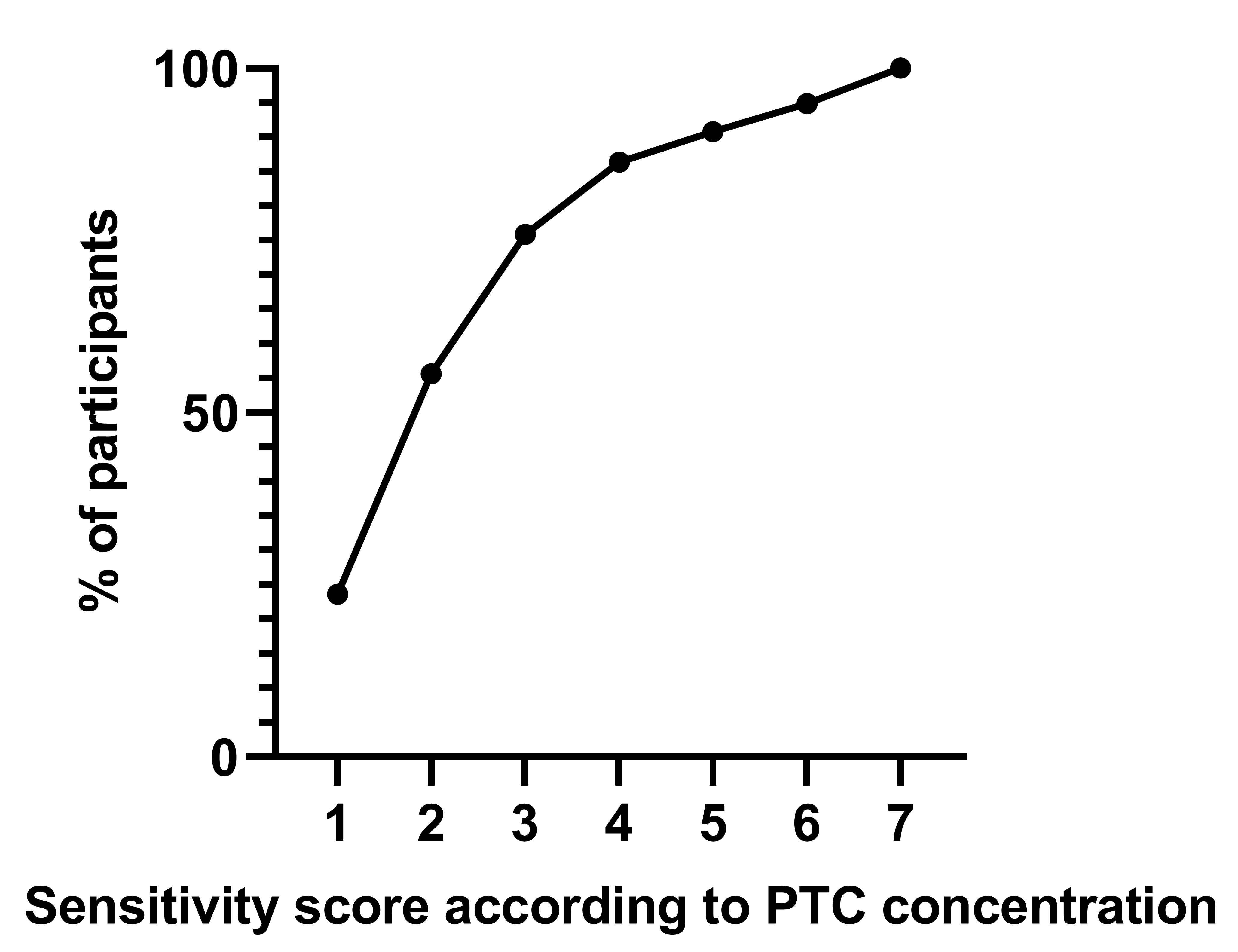


**Supplementary figure 1.** Cumulative frequency curves of the PTC recognition threshold scores.
